# Heterogeneous Recommendations for School Attendance in Children With Chronic Kidney Diseases During the COVID-19 Pandemic in Europe

**DOI:** 10.3389/fped.2021.646595

**Published:** 2021-03-03

**Authors:** Raphael Schild, Luke Hopf, Sebastian Loos, Jun Oh, Elena Levtchenko

**Affiliations:** ^1^University Children's Hospital, University Medical Center Hamburg Eppendorf, Hamburg, Germany; ^2^Department of Pediatric Nephrology and Organ Transplantation, University Hospitals Leuven, Leuven, Belgium; ^3^Department of Development and Regeneration, Katholieke Universiteit Leuven, Leuven, Belgium

**Keywords:** COVID-19, school, chronic kidney disease, children, immunosuppression, kidney transplantation, pediatric CKD

## Abstract

**Introduction:** After worldwide closures due to the COVID-19 pandemic, schools have reopened in most European countries in late 2020. Consequently, for children with chronic diseases the risks of COVID-19 have to be weighed against the long-time risks of missing school.

**Methods:** To evaluate the impact of chronic diseases on school attendance for children in Europe during the COVID-19 pandemic we conducted a survey among members of the European Society for Pediatric Nephrology (ESPN) between September and November 2020. We asked for current forms of schooling, the existence of national guidelines, parental concerns, and the pediatric nephrologists recommendations for school attendance for specific virtual patients with chronic kidney disease (CKD).

**Results:** Recommendations varied widely among pediatric nephrologists. A minority stated that specific recommendations for COVID-19 risk in children with kidney diseases existed in their country from local health authorities (9 of 29 countries; 31%) and/or national pediatric nephrology societies (9 of 29 countries; 31%). Over 90% of physicians have experienced parents keeping their children out of school against medical advice of their health providers and about 50% have experienced their patients being refused by school authorities. Consequently, 25% of all pediatric nephrologists estimated that more than 10% of their patients will not attend school regularly.

**Conclusion:** COVID-19 causes educational deficits in the already vulnerable population of children with CKD. As the evidence for the course of COVID-19 in children with chronic diseases grows, rapidly adapted recommendations from pediatric societies could help reduce uncertainty among doctors, patients, and parents.

## Introduction

The COVID-19 pandemic has led to widespread closing of schools during the first European outbreak in spring 2020. Since then the risk posed by school children as drivers of the pandemic has been a topic of discussion. In addition, while children generally do not seem to be at risk for a severe course of COVID-19 it is still unclear whether this also applies to children with chronic disease (1, 2). In the past months, schools and kindergartens have reopened and national shutdown strategies are aimed to keep them open at all costs in most European countries. Many governments have justified their decision by the burden that homeschooling had been putting on families. As a consequence, doctors, patients, parents, and schools have to decide which children are at risk and should be kept from school in the face of increasing infection rates. To investigate the different approaches in European countries for children with chronic kidney diseases we have conducted a survey among the members of the European Society for Pediatric Nephrology (ESPN).

## Methods

On behalf of the ESPN, we conducted an online survey among European pediatric nephrologists. The survey consisted of 14 questions regarding school attendance in pediatric patients with kidney diseases during the Sars-CoV2 pandemic. The survey was sent to all 604 ESPN members between September and October 2020. As no individual patient data were collected in this study, no ethics vote was obtained.

The questions included 13 closed and one open-ended question on profession, institution, number of patients treated, current school form at the start of the school term (homeschooling, regular classroom teaching, hygienic precautions) and the existence of national recommendations or guidelines for COVID-19 risk in children with chronic kidney diseases. To assess, which conditions pediatric nephrologists consider to pose a higher risk for a severe course of COVID-19, multiple clinical scenarios were presented. The physicians were asked to choose whether they would advise against attendance of school or kindergarten for these children. In order to assess the usual practice of school attendance in immunosuppressed children we asked how long children are usually kept out of school after kidney transplantation in the corresponding center. Response types included multiple choice and free text.

To assess whether the existence of guidelines was associated with differences in the physician's advice on school attendance in their patients we compared answers between the corresponding subgroups. A review of all available guidelines provided by participants for children with chronic kidney disease (CKD) was performed to sum up the core recommendations. Descriptive statistics were performed with MS Excel. DeepL Pro Version 1.15.0 was used to translate national guidelines.

## Results

We obtained 69 filled out questionnaires from 61 pediatric nephrology centers from 29 countries. Almost all (94%) of the participants were pediatric nephrologists. In a majority of countries physicians indicated that there are guidelines or recommendations from national health authorities on which patients with chronic illnesses are at increased risk for a severe course of COVID-19 disease [23 (79%) of 29 countries; 67% of physicians surveyed; Table 1]. However, for pediatric patients with kidney disease specific guidelines existed only in a minority of countries [13 (42%) of 29 countries], either from local health authorities [9 (31%) of 29 countries; 28% of physicians], or national pediatric nephrology societies [9 (31%) of 29 countries; 38% of physicians]. An overview of guidelines that were available and could be translated is given in Table 2. Only 28% of doctors surveyed stated that local education authorities and schools offer specific recommendations on which diseases should lead to exclusion from attendance in class.

**Table 1:** Survey Questions on the existence of COVID-19 guidelines and the assessment of COVID-19 related risks of attendance of school or kindergarten in CKD children.

**Existence of guidelines**					
Are there guidelines or recommendations in your home country…	yes				
	% of doctors surveyed			% of countries	
From the *local health authorities* on which *general medical conditions* lead to an increased COVID-19 risk?	67%			83%	
From the *local health authorities* on which *specific kidney diseases in children* lead to an increased COVID-19 risk?	28%			31%	
From *your national pediatric nephrology society* on which *specific kidney diseases in children* lead to an increased COVID-19 risk?	38%			31%	
**Assessment of COVID-19 related risks for clinical scenarios**					
Which of the following patients would you *exclude from school or kindergarten* under the circumstances currently present in your home country?	School (7–18 yrs)			Kndergarten (0–6 yrs)	
*Nephrotic Syndromes and other glomerulopathies*	n (%) of doctors surveyed				
Nephrotic syndrome, relapsing but no maintenance immunosuppression (currently in remission)	1 (1%)			1 (1%)	
Nephrotic syndrome, relapsing but no maintenance immunosuppression (currently active disease)	24 (35%)			25 (36%)	
Frequently relapsing nephrotic syndrome on immunosuppression (currently in remission)	12 (17%)			12 (17%)	
Frequently relapsing nephrotic syndrome on immunosuppression (currently active disease)	44 (64%)			45 (65%)	
Steroid resistant nephrotic syndrome on immunosuppression (in remission)	15 (22%)			15 (22%)	
Steroid resistant nephrotic syndrome on immunosuppression (active disease, no remission)	39 (57%)			40 (58%)	
Other glomerular diseases on immunosuppression e.g., Lupus nephritis, C3 Glomerulopathy (in remission)	12 (17%)			13 (19%)	
Other glomerular diseases on immunosuppression e.g., Lupus nephritis, C3 Glomerulopathy (active disease, no remission)	39 (57%)			36 (52%)	
*CKD*					
Chronic Kidney Disease (CKD) Stage 1	1 (1%)			1 (1%)	
Chronic Kidney Disease (CKD) Stage 2	1 (1%)			1 (1%)	
Chronic Kidney Disease (CKD) Stage 3	2 (3%)			3 (4%)	
Chronic Kidney Disease (CKD) Stage 4	9 (13%)			12 (17%)	
Chronic Kidney Disease (CKD) Stage 5 or 5D	23 (33%)			25 (36%)	
*Kidney transplantation*					
Patient < 3 months after Kidney transplantation (KTX)	62 (90%)			62 (90%)	
Patient > 3 months after KTX on maintenance immunosuppression	14 (20%)			15 (22%)	
Patient > 3 months after KTX on elevated immunosuppression (e.g., due to rejection treatment)	45 (65%)			43 (62%)	
*Other immunosuppression*					
aHUS on Eculizumab	19 (28%)			21 (30%)	
Patients on Rituximab (B cell depletion)	31 (45%)			30 (43%)	
Patients < 2 yrs after Rituximab treatment (B cells recovered)	0 (0%)			1 (1%)	
*Hypertension and other*					
Hypertension no end organ damage	0 0%			1 (1%)	
Hypertension with mild to moderate left ventricular hypertrophy	4 6%			4 (6%)	
Other conditions not mentioned here that you would exclude from school attendance: (free text)*	6 (9%)			4 (6%)	
**Decisions of parents and school authorities**					
	“No, never”		“Yes, but rarely”		“Yes, regularly”
Do schools sometimes refuse to include your patients in classroom teaching against your advice?	52%		43%		4%
Do parents sometimes refuse to send their children to school against your advice?	7%		67%		26%
Estimation of overall school attendance					
**Estimation of overall school attendance**					
How many of your patients *did not or will not* attend school regularly at the beginning of this school term?	“None or close to none”	“up to 10%”	“0–30%”	“30–50%”	“over 50%”
	26%	49%	16%	7%	1%

* *Other conditions filled out as free text were mostly patients with high intensity of immunosuppression (IV cyclophosphamide, methylprednisolone, ATG)*.

**Table 2:** Selection of recommendations for school attendance in children with kidney diseases from national pediatric or pediatric nephrology societies across europe.

**Country**	**Organization**	**Recommendations**	**Web resource**
Belgium	Sciensano (public health institution) COVID-19 pediatric scientific committee on schooling of children with comorbidities	No school attendance: - Corticosteroids > 20 mg/day prednisolone (or > 0.5 mg/kg/day for children < 40 kg) School attendance only after doctors advice: - Other immunosuppressive (combination) therapy, Rituximab, Eculizumab - Multiple serious disease	https://covid-19.sciensano.be/nl/covid-19-procedures
France	Société Française de Pédiatrie (French Pediatric Society) Société de Néphrologie Pédiatrique (SNP)	No general advice against school attendance In severe cases, individual decision of pediatrician recommended	https://www.snephroped.org/
The Netherlands	NVK (Dutch Pediatric Society)	No general advice against school attendance In severe cases, individual decision of pediatrician recommended	https://www.nvk.nl/
Spain	Spanish Association of Pediatrics Spanish Society of Pediatric Nephrology	School attendance with FFP2 mask: - Maintenance immunosuppression for any reasonNo school attendance: - Corticosteroids > 20 mg/day - 6 weeks after high dose corticosteroids - Rituximab, iv Cyclophosphamide, or other high dose immunosuppressants	https://www.aeped.es
United Kingdom	British Association of Pediatric Nephrology (BAPN) Royal College of Pediatrics and Child Health (RCPCH)	The following patients are classified as clinically extremely vulnerable (CEV) and should be kept from school during times when there is a general governmental advice for shielding of vulnerable patients: - Kidney transplantation in the last 3 months - For 4 weeks after starting high dose corticosteroids (> 20 mg per day) together with another immunosuppressant	https://renal.org/health-professionals/covid-19/bapn-resources https://www.rcpch.ac.uk/key-topics/covid-19

The assessment of pediatric nephrologists regarding the suitability of children and adolescents with chronic kidney disease for school attendance varied greatly (Table 1). Five percentage of the doctors would recommend school attendance for all the patients mentioned, slightly fewer excluded practically all patients in the virtual clinical scenarios from school. However, patients < 3 months after kidney transplantation were almost uniformly deemed unsuitable for school attendance under the current circumstances (90%). Patients on elevated immunosuppression after kidney transplantation (e.g., after rejection treatment) and patients with active glomerular disease on immunosuppression were also frequently excluded from school (Table 1). There were no obvious differences in these recommendations for school children vs. pre-school children. Moreover, when comparing answers of doctors in countries with or without COVID-19 guidelines for specific kidney diseases in children we did not find any significant differences in the assessment of the clinical scenarios. When asked about routine care after kidney transplantation outside of the pandemic, 35% of participants stated that they would normally allow their patients to attend school 2 months after surgery or less.

About half of all doctors surveyed said they had experienced their patients being denied school attendance by the schools, even though the doctors themselves had no medical objections to school attendance. However, only 4% of doctors stated that this was a regular practice. In contrast, more than 90% of physicians have experienced that parents, against their own advice, have kept their children out of school due to the COVID-19 threat. Twenty six percentage of doctors surveyed said that this happens regularly.

The estimation of how many of their patients in total would be unable to attend school at the beginning of the school year in mid 2020, either because of medical advice, parental concerns or rejection by the school, was variable among the doctors surveyed. About a quarter of them estimated that 10 per cent or more of their patients did not or will not attend school. This statement was more frequent among physicians from countries with existing pediatric kidney specific guidelines compared to those without guidelines (35 vs. 15%). On the other end of the spectrum, a quarter of the pediatric nephrologists stated that almost all their patients were attending school.

Classroom teaching under hygiene protection measures was by far the most common type of teaching performed at the time of this survey (68%). Few participants stated that only regular classroom teaching took place (6%). Homeschooling was available in addition to classroom teaching in 20% of cases but only in one center schools were completely closed and homeschooling was the only form of schooling.

## Discussion

In our survey we found that in the majority of European countries there are no specific recommendations on school attendance for children with CKD in the COVID-19 pandemic. Consequently, there is a wide variability in the advice pediatric nephrologists give their patients. A minor but significant group of children with CKD will not attend school regularly during this school term.

While the risk of a severe course of COVID-19 is markedly increased in adults with CKD (3, 4) recent evidence on children with CKD or under immunosuppression is encouraging and points toward a milder course in this population (5–8). However, among reported severe cases and deaths in children with COVID-19, preexisting comorbidities are a frequent finding (1, 9). Therefore, a higher risk is assumed for some children with CKD by most physicians, but a high degree of uncertainty remains. This is reflected by the variability of the physician's assessment in our survey. Most physicians decide on a case by case basis, assessing the risk of a severe course of COVID-19 in each individual patient based on the underlying disease, comorbidities and immunosuppressive medication. Moreover, the decision has to be made together with the parents and patients, who might differ in their assessment of the risks and benefits. Consequently, our survey shows that most of the pediatric nephrologists have experienced parents weighing the risks of COVID-19 higher. However, whether parents are generally more concerned than doctors on this issue, was not captured by our survey. In addition to the lack of evidence, the high variability in the assessment of the risk situation may be due to differences in the conditions present at the time of the survey, especially the local infection rates and the confidence of parents and doctors in the schools' corona protection measures.

School closures have far-reaching effects on the psychological development of children and young people. Furthermore, there is evidence that reduced educational attainment is associated with a lower life expectancy and that the school closures during the COVID-19 pandemic could therefore result in a loss of life years for the children affected (10). This has a particularly large impact on chronically ill or disabled children, since they are most likely to be further disadvantaged after school reopenings by not being able to attend school due to health concerns (11). International or regional pediatric nephrology societies like the IPNA and ESPN therefore could play a key role in promoting child development and child well-being by providing guidance during pandemics.

Our review of existing national guidelines is showing that recommendations for school attendance in kidney disease mainly focus on patients with high levels of immunosuppression (Table 2). However, in our survey a significant fraction of physicians tended to be more cautious and exclude patients that would have been allowed to attend school under most of these recommendations. Interestingly, the replies of participants from the same countries about the existence of guidelines were often contradictory. This could indicate that even within those countries the recommendations are not always known or followed by the treating physicians. Therefore, evidence based European recommendations might help reduce uncertainties among doctors and allow more children with kidney diseases to attend school.

From our own experience, personal communications and our review of the most recent literature and guidelines on COVID-19 in children it seems reasonable to consider only those children with kidney disease at risk for a severe course of COVID-19 who are on at least a dual immunosuppressive therapy including currently or recent high doses of steroids (such as prednisone > 20 mg per day or 30 mg/m^2^ per day). In practice, this applies to children during the first 3 months after KTX, after rejection treatment or during immunosuppressive induction therapy for other reasons. This is mostly in line with the excellent guidance provided by the Royal College of Pediatrics and Child Health and the British Academy of Pediatric Nephrology (Table 2). Of note, these recommendations are cautious as there is still is no clear evidence that even these children are at a higher risk and most of the data is derived from adult studies (5–9). However, if followed consequently this should allow the majority of children with kidney disease to attend school even in times when general shielding is advised.

Our study has several limitations. Since the survey reflects estimations of the physicians, we cannot make a statement on the exact prevalence of school attendance in children with CKD. Our review of existing guidelines might be incomplete since not all guidelines might have been reported. Furthermore, some guidelines are frequently updated and might have been slightly different at the time the person answered the survey.

## Conclusion

Only a minority of European countries have issued guidelines on school attendance for children with kidney disease. Consequently, pediatric nephrologists differ in their recommendations for their patients. Therefore, even though school closures and homeschooling will help to prevent spreading effects of the virus and will save lives, they place a heavy burden on children and families with regard to development and education. While our study looked at children with chronic kidney disease, this applies to all children with chronic disease states. Evidence based European or international guidelines could help reduce uncertainty and improve access to classroom teaching for chronically ill children. Since the COVID-19 pandemic is an emerging, rapidly evolving situation with almost daily changing of our knowledge of this serious infectious disease it is very comprehensible that generally valid recommendations are difficult to develop. They need to be constantly adapted and improved. Nevertheless, governments and medical societies should try to provide evidence-based guidelines for special at-risk populations, even when evidence is scarce, because especially in such difficult and frightening times people need guidance and clear messages.

## Data Availability Statement

The raw data supporting the conclusions of this article will be made available by the authors, without undue reservation.

## Author Contributions

RS has been involved in study design, study conduct, data analysis, and the writing of the manuscript. LH and SL have been involved in literature search, evaluation of current guidelines and in co-writing, and revising the manuscript. JO and EL have been involved in the design and conduct of the study as well as revising the manuscript. All authors contributed to the article and approved the submitted version.

## Collaborators

Members of the ESPN: Serena Abbate, Marina Aksenova, Justine Bacchetta, Sevcan Bakkaloglu, Ortraud Beringer, Anna Bjerre, Olivia Boyer, Per Brandström, Ana Castellano, Martin Christian, Elisabeth Cornelissen, Cécile Dau, Katja Doerry, Jan Dudley, Ismail Dursun, Ruhan Düşünsel, Francesco Emma, Laura Espinosa, Marc Fila, Yaacov Frishberg, Mihai Gafencu, Blazquez Gomez, Ryszard Grenda, Deirdre Hahn, Nakysa Hooman, Dmitry Ivanov, Timo Jahnukainen, Augustina Jankauskiene, MatjaŽ Kopač, Rachel Lennon, Max Liebau, Jacques Lombet, Mercedes Lopez Gonzalez, Javier Lumbreras Fernández, Nataša Marčun Varda, Matko Marlais, Daniela Marx-Berger, Drew Maxted, Heather Maxwell, Marta Melgosa, Kristina Moeller, Giovanni Montini, Hulya Nalcacioglu, Lars Pape, Ludmila Podracka, Larisa Prikhodina, Nikoleta Printza, Anna Raes, George Reusz, Abdalla Saghar, Fernando Santos, Michiel Schreuder, Hadas Shasha-Lavsky, Graham Smith, Stella Stabouli, Natasa Stajic, Malgorzata Stanczyk, Hagen Staude, Maria Szczepańska, Ana Teixeira, Julia Thumfart, Rezan Topaloglu, Despina Tramma, Roos van Rooij, Lutz T. Weber, Irith Weissman, Marcus Weitz, Louise Winding, and Natalia Zaikova.

## Conflict of Interest

The authors declare that the research was conducted in the absence of any commercial or financial relationships that could be construed as a potential conflict of interest.

## References

[1] Lanyon N du Pre P Thiruchelvam T Ray S Johnson M Peters MJ. Critical paediatric COVID-19: varied presentations but good outcomes. Arch Dis Child. (2020) 1–2. 10.1136/archdischild-2020-319602 32601086

[2] Gotzinger F Santiago-Garcia B Noguera-Julian A Lanaspa M Lancella L Calo Carducci FI. COVID-19 in children and adolescents in Europe: a multinational, multicentre cohort study. Lancet Child Adolesc Health. (2020) 4:653–61. 10.1016/S2352-4642(20)30177-2 32593339 PMC7316447

[3] Valeri AM Robbins-Juarez SY Stevens JS Ahn W Rao MK Radhakrishnan J. Presentation and outcomes of patients with ESKD and COVID-19. J Am Soc Nephrol. (2020) 31:1409–15. 10.1681/ASN.2020040470 32467113 PMC7350989

[4] Nair V Jandovitz N Hirsch JS Nair G Abate M Bhaskaran M. COVID-19 in kidney transplant recipients. Am J Transplant. (2020) 20:1819–25. 10.1111/ajt.15967 32351040 PMC7267603

[5] Goss MB Galvan NTN Ruan W Munoz FM Brewer ED O'Mahony CA. The pediatric solid organ transplant experience with COVID-19: an initial multi-center, multi-organ case series. Pediatr Transplant. (2020) e13868. 10.1111/petr.13868 32949098 PMC7537006

[6] Marlais M Wlodkowski T Vivarelli M Pape L Tonshoff B Schaefer F. The severity of COVID-19 in children on immunosuppressive medication. Lancet Child Adolesc Health. (2020) 4:e17–8. 10.1016/S2352-4642(20)30145-0 32411815 PMC7220160

[7] Melgosa M Madrid A Alvarez O Lumbreras J Nieto F Parada E. SARS-CoV-2 infection in Spanish children with chronic kidney pathologies. Pediatr Nephrol. (2020) 35:1521–4. 10.1007/s00467-020-04597-1 32435879 PMC7237873

[8] Plumb L Benoy-Deeney F Casula A Braddon FEM Tse Y Inward C. COVID-19 in children with chronic kidney disease: findings from the UK renal registry. Arch Dis Child. (2020) 1. 10.1136/archdischild-2020-319903 32709685

[9] Oualha M Bendavid M Berteloot L Corsia A Lesage F Vedrenne M. Severe and fatal forms of COVID-19 in children. Arch Pediatr. (2020) 27:235–8. 10.1016/j.arcped.2020.05.010 32518045 PMC7269941

[10] Christakis DA Van Cleve W Zimmerman FJ. Estimation of US children's educational attainment and years of life lost associated with primary school closures during the coronavirus disease 2019 Pandemic. JAMA Netw Open. (2020) 3:e2028786. 10.1001/jamanetworkopen.2020.28786 33180132 PMC7662136

[11] Joline EB Lainie KH Susan DA Amy JH Robert R Maurice GS. School reopening during COVID-19 pandemic: considering students with disabilities. J Pediatr Rehabil Med. (2020) 13:425–31. 10.3233/PRM-200789 33136082

